# Serum Profiles of Cytokines in Behcet’s Disease

**DOI:** 10.3390/jcm6050049

**Published:** 2017-05-02

**Authors:** Alireza Sadeghi, Fereydoun Davatchi, Farhad Shahram, Arezoo Karimimoghadam, Majid Alikhani, Aiyoub Pezeshgi, Saeideh Mazloomzadeh, Bahar Sadeghi-Abdollahi, Masoud Asadi-Khiavi

**Affiliations:** 1 Department of Internal Medicine, School of Medicine, Zanjan University of Medical Sciences, Zanjan 4515613191, Iran; sadeghi_alireza50@yahoo.com (A.S.); arezookarimimoghaddam@yahoo.com (A.K.); majid.alikhani1981@gmail.com (M.A.); 50ayoub@zums.ac.ir (A.P.); 2 Department of Internal Medicine, School of Medicine, Tehran University of Medical Sciences, Tehran 3439123900, Iran; fddh@neda.net (F.D.); shahramf@tums.ac.ir (F.S.); baharsadeghi@gmail.com (B.S.-A.); 3 Social Determinants of Health Research Center, Zanjan University of Medical Sciences, Zanjan 7797845157, Iran; smazloomzadeh@zums.ac.ir; 4 Zanjan Applied Pharmacology Research Center, Zanjan University of Medical Sciences, Zanjan 4513956184, Iran; 5 Department of Pharmacotherapy, School of Pharmacy, Zanjan University of Medical Sciences, Zanjan 4513956184, Iran

**Keywords:** cytokines, Behcet’s disease, uveitis

## Abstract

**Introduction:** Behcet’s disease (BD) is a chronic systemic autoinflammatory vasculitis which is handled by the variety of proteins like cytokines. Therefore, cytokines are considered as one of the prototypic factors during inflammatory responses of BD. Consequently, the present study was designed for evaluation of cytokine profiles in Iranian BD cases, including those with and without uveitis. **Materials and Method:** All cases were divided into three groups based on ophthalmologic exam results: BD with uveitis, BD without uveitis, and recovered uveitis BD. Cases with a history of BD recovery were placed in the group of recovered uveitis. The patients with infectious uveitis as well as other collagen vascular diseases and patients who have used biologics to treat ocular immune-mediated diseases were excluded. Finally, after venous blood sampling, levels of cytokines were quantified and statistical approaches were performed for measurements. **Results:** Enrolled cases were divided to 26 patients with active uveitis, 25 patients with recovered uveitis and 24 patients without uveitis and interestingly, just IL-2 was the only cytokine that showed statistical difference in patients with BD uveitis in comparison with other groups (*p*_value_ = 0.02). The pair wise comparison showed a significant difference between the patients with and without uveitis groups (*p*_value_ = 0.004) as well as patients with uveitis and recovered uveitis groups (*p*_value_ = 0.002). **Discussion:** Significant elevation of IL-2 in patients with uveitis (in comparison with recovered or without uveitis cases) demonstrates that it may be one of the main proteins that enroll in the pathophysiology of BD uveitis and may be considered as a new target for refractory disease therapies. Studies with larger samples can help to obtain more accurate conclusions.

## 1. Introduction

Behcet’s disease (BD) is a chronic disorder, and its manifestations are thought to originate from autoinflammatory-based vasculitis. Inflammation can also derive from arthropathies and vasculopathies in the central nervous system [1,2]. Diagnosis of this syndrome is based on clinical aspects and internationally accepted criteria. This syndrome affects young men and women in the Mediterranean, the Middle East, and the Far East regions, which reveals the possibility of a relationship between the ancient Silk Road regions and the prevalence of the BD. This syndrome affects male and female equally but severe cases occur more in males [3]. The main pathological view shows systemic perivasculitis with neutrophilic infiltration and endothelial cell swelling. A large number of infiltrative CD4+ T cells as well as neutrophils are also seen in lesions [4]. Increases or changes in the levels of certain cytokines in BD has been demonstrated in many studies [5,6,7]. BD is known as autoimmune disease due to increased levels of immunoglobulins, immune complexes, and acute phase proteins [8]. Abnormality in endothelial cells and neutrophils is assumed as the starting points for many clinical manifestations of BD. Due to the activation of the immune system, cytokines, and inflammatory mediators can modulate the disease process [9]. BD uveitis is a chronic uveitis associated with necrotizing vasculitis in the retina, and it can give rise to bilateral blindness that is needed for heavy immunosuppression. Recent studies have shown that immune factors in BD uveitis are distinguishable from the other endogenous uveitis [5,10]. The association between cytokines and uveitis has been already mentioned in a limited number of studies, but their methods of implementation or analysis of cytokines have been partly imperfect [11,12,13]. Thus, it is desirable to perform an appropriate implementation method as well as evaluating more number of cytokines particularly in the case of uveitis as one of the most terrifying status in BD.

## 2. Materials and Method

This study was designed to evaluate biomarkers and cytokines entitled VEGF, IFNγ, TNFα, IL-17, IL-15, IL-12, IL-10, IL-8, IL-6, IL-4, and IL-2 in venous blood samples of patients who divided in three groups and handled by subsequent statistical approach. This survey was done on patients referred to a rheumatology clinic in a university hospital in Zanjan, Iran. The research followed the tenets of the Declaration of Helsinki; detailed design of the study was explained to the patients and their families and/or legal guardians. Written informed consent was obtained from all subjects and their legal executors. Ethical permission was obtained, all information remained confidential and mural aspects of study were accepted in details of the plan adopted in the Medical Ethics Committee of Zanjan University of Medical Sciences. Ethical issues (including plagiarism, misconduct, data fabrication, falsification, double publication or submission, and redundancy) have been completely observed by the authors.

Patients with confirmed diseases based on New International Criteria for BD (ICBD) were divided in three groups according to the ophthalmologic exam results: BD with uveitis, BD without uveitis, and recovered uveitis BD. Patients with a post-recovery history of BD uveitis for at least 3–6 months were placed in the group entitled “recovered uveitis BD”. The patients with infectious uveitis, other collagen vascular diseases and biologic drug users were excluded as well. Age, sex, and Pathergy test results were obtained for each patient as well. A quantity of 10 ml of venous blood was taken and was frozen at −70 °C after centrifugation at 3000 rpm to separate the serum. Subsequently, collected samples were conducted for evaluation of VEGF, IFNγ, TNFα, IL-17, IL-15, IL-12, IL-10, IL-8, IL-6, IL-4, and IL-2 levels using Enzyme Linked Immunosorbent assay (ELISA) method via kits (Bioassay Technology Co., Shanghai, China). The test sensitivity of each corresponding kits were 2.33, 2.52, 1.02, 2.43, 1.13, 2.52, 1.98, 0.98, 1.52, 0.49, and 10.25 ng/L respectively. Intra assay precision (CV < 10%) and Inter assay precision (CV < 12%) were done using another ELISA processor. Analysis of variance (ANOVA) and Kruskal–Wallis tests were used for quantitative variables with normal distribution and for non-normally distributed variables respectively. Additionally, Bonferroni correction test and Mann–Whitney U post-hoc test were done separately to weigh against the two groups.

## 3. Results

Seventy-five patients participated in three groups based on clinical examination and already-mentioned diagnostic criteria (Table 1). The following results were obtained after measurement of serum cytokines and analyzed them in all three groups (Table 2). Results of data analysis of IL-2 serum levels in the three groups were as follows: BD without uveitis (mean = 537, SD = 568), BD with uveitis (mean = 1184, SD = 1658), and recovered uveitis BD (mean = 506, SD = 491), respectively. Additionally, due to the non-normal distribution of data, the median was calculated to balance three groups: BD without uveitis (median = 376), BD with uveitis (median = 542), and recovered uveitis BD (median = 365). Significant differences in IL-2 serum levels were observed among groups after a median comparison (*p*_value_ = 0.02).

The Mann–Whitney U test showed significant differences between IL-2 serum levels of groups entitled “BD with uveitis” and “recovered uveitis BD” (*p*_value_ = 0.002). A significant difference in IL-2 serum levels between the “BD with uveitis” group and the “BD without uveitis” group was also observed (*p*_value_ = 0.004), but no significant difference was observed between “BD without uveitis” and “recovered uveitis BD” groups (*p*_value_ = 0.47). No significant differences were observed among other items.

## 4. Discussion

Results showed that only IL-2 increased in cases of active uveitis, and, after treatment (without any using of biologic agents), it decreased in cases of recovered uveitis BD. Other cytokines did not show any significant changes in the three groups, so its pathogenic role in BD with uveitis was not proven in our study. The increase of IL-2 in BD patients with active uveitis and the decrease in BD cases without uveitis and recovered BD uveitis could be evidence for the role of CD4**^+^**T helper cells in patients with uveitis [12,13]. Because only 40% of BD patients with active uveitis were enrolled in the study, there was no statistical difference between IL-2 levels in the BD uveitis group compared to uveitis cases in the study of Nalbant et al. [14]. Our findings showed results that were identical to Sugiet et al.’s study with similar findings with respect to IL-2 levels in cases of BD uveitis [15]. IL-4, as an anti-inflammatory cytokine, showed no significant differences in any of the three groups. Levels of this cytokine were lower in patients with BD uveitis in comparison with the control group in the study of Nalbant et al. However, as mentioned already, only 40% of patients had active uveitis in our study, which could be considered as a confounding factor in the case of both studies comparison. The findings were different with respect to IL-4 in Sugiet et al.’s study and showed no significant differences in patients with BD uveitis in comparison with the control group, which shows data identical to our findings. Although it has been shown that the activation of BD is associated with IL-6 as a pre-inflammatory cytokine [14,16], but its prominent role in involvement of central nervous system (CNS) due to cytokines elevated levels in cerebrospinal fluid of patients was proven in Wang et al.’s study [17]. However, IL-6, in our study, was identical to that in Nalbant et al.’s survey, and there were no significant differences in IL-6 levels among the groups [14]. Conversely, Bardak et al.’s study showed a disparity compared to Nalbant et al.’s and our studies. This means that IL-6 levels in BD uveitis in comparison with the control groups was reduced after treatments [18]. Perhaps the reason for this difference originates from the small sample size of the study. Previous studies have shown that IL-8 as an essential cytokine in angiogenesis and chemotaxis of neutrophils rises in the active phase of the BD [16]. There was no association between IL-8 and BD uveitis in our study, but such a relationship was shown in Durmazlar et al.’s study [19]. IL-8 was reduced in patients with active BD uveitis in Nalbant et al.’s study, but there was an elevation in active BD uveitis, and it was reduced during therapies in Bardak et al.’s study [18]. These findings are different from our results, which could be due to the small sample size. IL-10 levels as a Th_1_ cell inhibitor cytokine, showed no differences among the three groups in our study. However, an increase in IL-10 was only shown in aqueous humor in El-Asrar et al.’s study [20]. This story was repeated in Ahn et al.’s study as well [4]. Guenane et al.’s study showed the same results, but none of the samples of that study were directly obtained from the patient’s serum (because of using sera and supernatants of peripheral blood mononuclear cells cultures), and cases without BD uveitis were not studied. Additionally, a comparison was performed only between controls and idiopathic uveitis. IL-12 plays an important role as a classical cytokine of Th1 cells in patients with visceral organs inflammation. In contrast, a role for this cytokine was not shown in BD uveitis based on our findings. As a non-specific cytokine, increasing in the level of IL-12 were demonstrated in idiopathic uveitis and BD uveitis in Guenane et al.’s study, which shows its role in uveitis regardless of the underlying disease. These findings contrast with those of Ahn et al.’s study [4,21]. According to our findings, VEGF, TNFα, and IFNγ had no role in BD uveitis [4,14,21]. Furthermore, IFNγ could be considered as a non-specific cytokine in uveitis according to findings described in [22].

## 5. Conclusions and Suggestions

The significant elevation of IL-2 in patients with uveitis indicates that it is likely the main role in the pathophysiology of BD uveitis and may be considered as a new target for refractory disease therapies. Studies with larger samples can help to obtain more accurate conclusions.

## Figures and Tables

**Table 1:** Demographic and clinical conditions of enrolled cases.

Demographic and Clinical Variables		BD with Uveitis *n* = 26	Recovered Uveitis BD *n* = 25	BD without Uveitis *n* = 24
Age		38.6 ± 3.1	40.1 ± 3.3	36.5 ± 3.2
Gender	Female	5	11	6
	Male	21	14	18
Ocular lesion		26	25	-----
Aphthous stomatitis		25	24	22
Genital aphthosis		13	10	21
Skin lesion		20	18	14
CNS involvement		-----	-----	-----
Vascular manifestation		3	2	7
Arthritis		4	5	10
Pathergy test		12	12	9

**Table 2:** Levels of cytokines in venous blood samples of enrolled cases (μg/dL).

Variable	Variable		BD without Uveitis		BD with Uveitis		* *p*_vaule_
	Mean ± SD	Median (25–75%)	Mean ± SD	Median (25–75%)	Mean ± SD	Median (25–75%)	
IL_2_	568 ± 537	351–470 (376)	506 ± 491	347–423 (365)	1658 ± 1184	451–672 (542)	0.02
IL_4_	338 ± 262	152–223 (182)	296 ± 268	182–250 (200)	444 ± 363	181–266 (208)	0.12
IL_6_	100 ± 66	62–124 (62)	88 ± 64	56–71 (63)	90 ± 73	57–76 (62)	0.23
IL_8_	448 ± 334	150–338 (158)	435 ± 298	126–228 (164)	554 ± 394	117–200 (137)	0.14
IL_10_	156 ± 106	105–154 (123)	145 ± 94	108–134 (115)	167 ± 148	106–138 (117)	0.59
IL_12_	180 ± 161	95–190 (122)	182 ± 180	94–174 (114)	226 ± 217	95–214 (109)	0.89
IL_15_	248 ± 203	139–204 (157)	245 ± 205	139–180 (160)	286 ± 225	160–182 (167)	0.25
IL_17_	114 ± 101	56–102 (73)	100 ± 94	59–89 (67)	145 ± 108	59–79 (70)	0.97
TNFα	165 ± 120	114–179 (123)	172 ± 109	113–200 (127)	166 ± 142	111–152 (124)	0.77
VEGF	1156 ± 957	661–1396 (702)	1081 ± 920	622–1220 (696)	1028 ± 1024	592–890 (673)	0.51
IFNγ	115 ± 68	78–143 (81)	109 ± 68	74–97 (79)	104 ± 73	73–93 (80)	0.41

* *p*_value_ lower than 0.05 was considered statistically significant.
